# Molecular engineering of mechanophore activity for stress-responsive polymeric materials

**DOI:** 10.1039/c4sc01945h

**Published:** 2015-02-12

**Authors:** Cameron L. Brown, Stephen L. Craig

**Affiliations:** a Department of Chemistry , Duke University , Durham , NC 27708-0346 , USA . Email: stephen.craig@duke.edu ; Tel: +1 919 660 1538

## Abstract

Force reactive functional groups, or mechanophores, have emerged as the basis of a potential strategy for sensing and countering stress-induced material failure. The general utility of this strategy is limited, however, because the levels of mechanophore activation in the bulk are typically low and observed only under large, typically irreversible strains. Strategies that enhance activation are therefore quite useful. Molecular-level design principles by which to engineer enhanced mechanophore activity are reviewed, with an emphasis on quantitative structure–activity studies determined for a family of *gem*-dihalocyclopropane mechanophores.

## Abstract 2

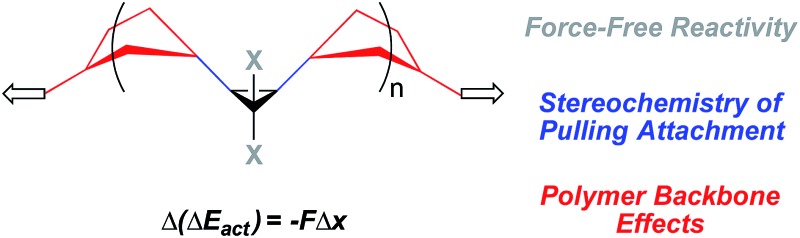
Molecular-level design principles by which to engineer enhanced mechanophore activity are reviewed, with an emphasis on quantitative structure–activity studies determined for a family of *gem*-dihalocyclopropane mechanophores.

## Introduction

**Fig. 1:**
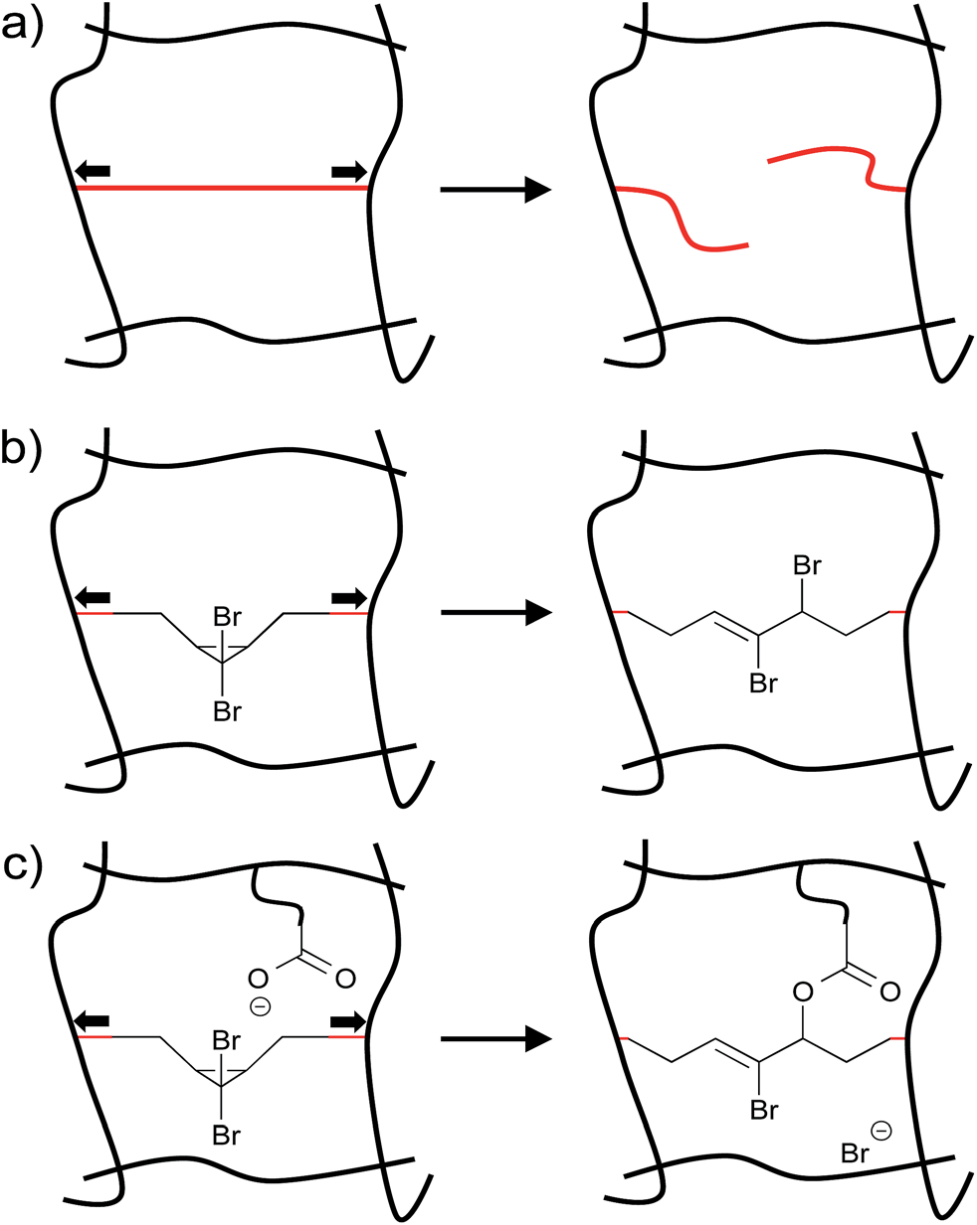
(a) Stress in polymers localize along individual polymer subchains, resulting in chain scission that can lead to material failure. Mechanophores can be embedded within these overstressed subchains to trigger a constructive, rather than a destructive response. For example: (b) *gem*-dibromocyclopropane will ring open in response to high forces of tension, releasing stored length that provides local stress relief in the overstressed chains, and (c) the 2,3-dibromoalkene products of the ring opening are cross-reactive toward mild nucleophiles such as carboxylates, and that reactivity has been exploited to generate *in situ* cross-linking and stress-strengthening.

Materials often fail as a result of the mechanical loads they experience during use.^1–4^ On the molecular level, forces within polymers are distributed unevenly throughout a material, and some polymer subchains experience greater stress than others.^5^ In some cases, the forces experienced by these overstressed subchains can trigger chain scission events (Fig. 1a). Chain scission in turn might nucleate the formation of a microcrack that subsequently propagates, ultimately leading to material failure.^6^ In recent years, force reactive functional groups, or mechanophores, have emerged as the basis of a potential strategy for not only signalling,^7–9^ but also combatting this destructive cascade. The strategy comprises embedding mechanophores along the polymer backbone or within cross-links, so that otherwise destructive forces within an overstressed subchain trigger a constructive, rather than a destructive, response.^2–4^

Examples of potentially constructive responses include the activation of latent catalysts that cross-link the bulk polymer matrix,^10^ a framework within which a recently reported “mechanoacid” might be particularly useful.^11^ Stoichiometric approaches have recently shown promise as well. For example, *gem*-dibromocyclopropanes embedded within the backbones of poly(butadiene) based polymers will ring open in response to high forces of tension, releasing stored length that provides local stress relief in the overstressed chains (Fig. 1b).^12^ In addition, the 2,3-dibromoalkene products of the ring opening are cross-reactive toward mild nucleophiles such as carboxylates, and that reactivity has been exploited to generate *in situ* cross-linking and order-of-magnitude strengthening in bulk polymers exposed to the typically destructive shear forces of twin-screw extrusion (Fig. 1c).^13^

**Fig. 2:**
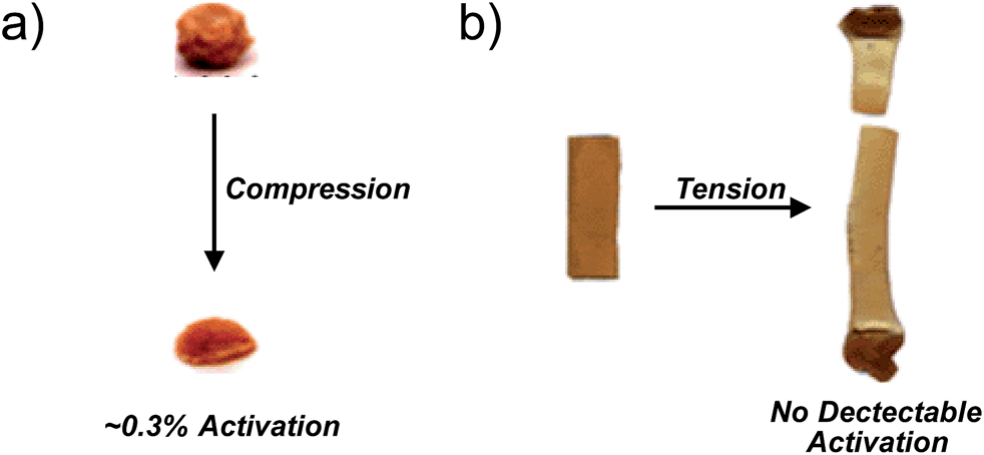
(a) Compression mechanically activates the *g*DBC, but only very low levels of activation are observed despite the dramatic, irreversible deformation of the bulk material. (b) Tensile strain applied to a *g*DBC–poly(butadiene) cast film to the point of failure does not lead to detectable *g*DBC ring opening (by ^1^H NMR). Adapted from ref. 18 with permission from The Royal Society of Chemistry.

Among the challenges limiting the general utility of the mechanophore strategy is that the levels of mechanophore activation in the bulk are typically low and observed only under large, typically irreversible strains.^8,14–18^ The example of *gem*-dibromocyclopropane (*g*DBC) mechanophores embedded in poly(butadiene), referenced above, is instructive in this regard. Lenhardt *et al.* examined mechanophore response in these systems under unconstrained uniaxial compression, and found that only very low levels (approximately 0.3%) of embedded mechanophores are activated in response to 36 MPa of compression. Not surprisingly, these forces lead to dramatic, irreversible deformation of the bulk material, a ball of which is effectively squashed into a flat pancake of polymer (Fig. 2a).^18^ Uniaxial tension is even less effective, with no mechanophore activation detected by ^1^H NMR in films stretched to failure (Fig. 2b).^18^ Obviously, the low levels of activation and large extent of permanent deformation places a fundamental limit on the utility of the mechanophore approach, and so strategies that enhance activation are quite useful. Broadly, the problem can be divided into material-level approaches and molecular-level approaches. The former involves identifying those material architectures that efficiently funnel macroscopic forces to mechanophores in the absence of irreversible deformation, as has been demonstrated recently in elastomers,^20,21^ but the properties of the material itself can in general have a significant impact on the extent of activation. Such effects are obviously important, but not the focus of this review. The latter set of approaches involves engineering at the level of molecular structure the appropriate reactivity and structural connections to generate the desired response as a function of force. In particular, the following question can be posed: for a given mechanophore motif, what structural features dictate the force required for activation to occur on a given time scale? This mini-review focuses on this molecular-level question by summarizing recent work on the effect of various molecular structural perturbations on the activity of a mechanophore. The emphasis is on quantitative force–activity relationships, for which *gem*-dihalocyclopropane mechanophores serve as a valuable reference system that is highlighted throughout the review.

## Fundamentals of covalent polymer mechanochemistry

At the most general level, the fundamentals of covalent mechanochemistry are well established. The action of mechanical force effectively reduces the activation barrier (Δ*E*_act_) of a reaction, by coupling mechanical work to the nuclear motions associated with the reaction. The energy provided by the mechanical work is given by the applied force times the distance, Δ*x*, over which that force is applied as the reactant goes from its ground state to transition state. We discuss Δ*x* in more detail below, but it can be regarded as an activation length – the difference in nuclear position at the transition state relative to that of the reactant, projected along the vector of applied force, *F*. The energy provided by work need no longer be provided by thermal fluctuations, and so the required activation energy changes as

1 Δ(Δ*E*_act_) = –*F*Δ*x*

Note that eqn (1) does not explicitly consider the interdependence of *F* and Δ*x*. In covalent polymer mechanochemistry, an overstressed polymer chain typically delivers force to the mechanophore, and the relatively small geometry changes that accompany an individual reaction (Δ*x* ∼ 1 Å) have a negligible impact on both the extension of the polymer chain and, consequently, the coupled force. An assumption of constant *F* is therefore typically justified. On the other hand, the position of both the ground state and the transition state (and hence their force-coupled difference, Δ*x*) shift when coupled to an applied force, to the extent that at sufficiently high forces the force-free transition states of some reactions even become new global minima on the force-coupled potential energy surface.^22^ In general, Δ*x* is therefore a function of *F*, and this can be accounted for directly in computations by adding terms into the system Hamiltonian^23,24^ or by applying reasonable approximations in the form of truncated Taylor expansions^25,26^ or analytical forms for the potential energy surface.^27–30^ As it does not influence the main points of this mini-review, we do not consider the dependency of Δ*x* on *F* further, but we are mindful that it ultimately is at play in any mechanochemical reaction.

Following from eqn (1), the rate of a given mechanochemical reaction (*i.e.*, the activity of a given mechanophore) is therefore given by

2 *k*(*F*) ∝ e^–(Δ*E*_act_–*F*Δ*x*)/*RT*^

Eqn (2) captures the key features that should be considered when designing or evaluating a mechanophore: (i) the intrinsic, force-free reactivity of the mechanophore (Δ*E*_act_); (ii) the magnitude of the applied force (*F*); and (iii) how well that force is coupled to the reaction pathway (Δ*x*). This analysis applies to cases in which activity is under kinetic control, as opposed to circumstances in which displaced equilibria are at play.^14^ The question of “how much force is necessary” is therefore time scale dependent, and the time dependence is reflected in using a force-dependent rate constant *k*(*F*) as the measure of mechanophore activity.

**Fig. 3:**
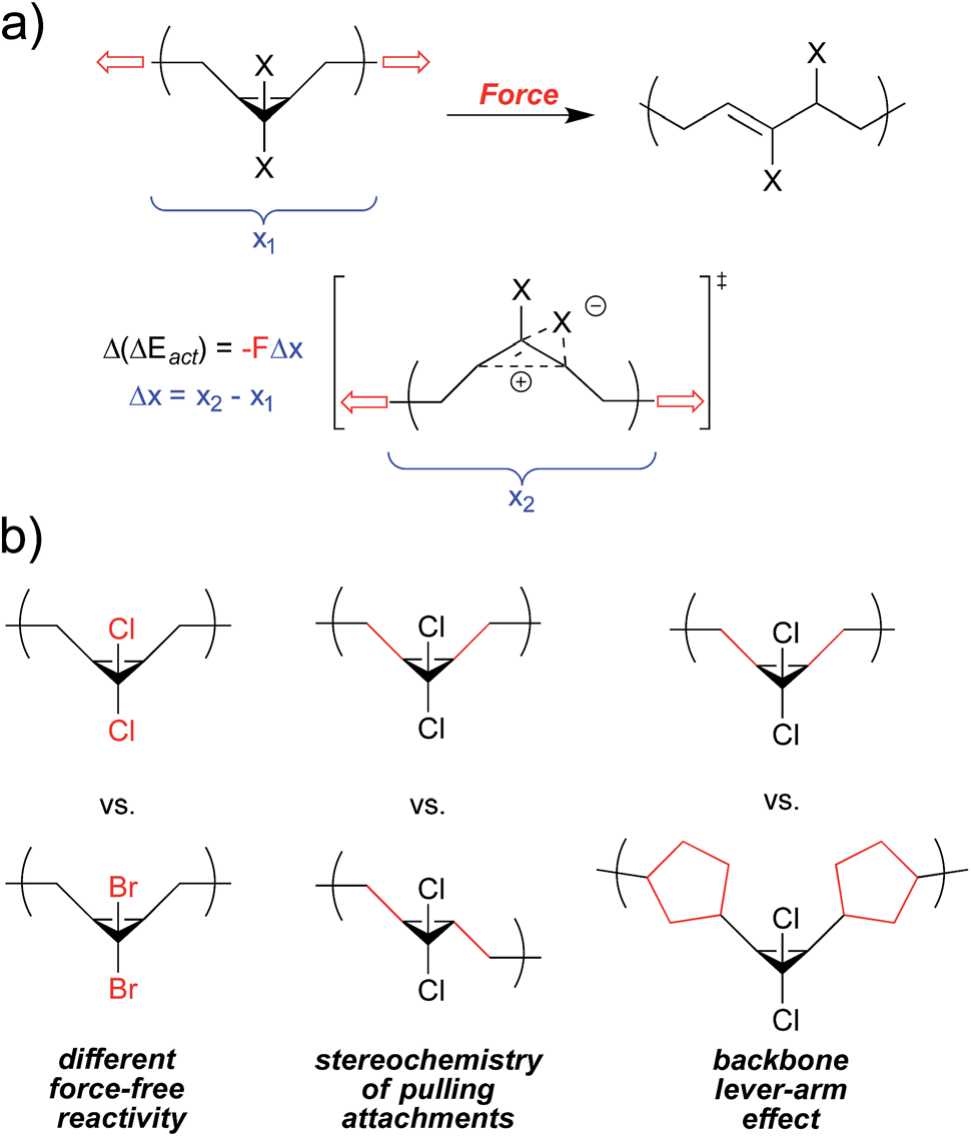
*gem*-Dihalocyclopropane undergoes a force-induced ring opening to a 2,3-dihaloalkene, and serves as a platform to quantitatively compare mechanical reactivity as a function of force-free reactivity, the geometry of attachment, and polymer backbone effects. The change in length along the vector of applied force on going from reactant to transition state, Δ*x*, is depicted here as a local change, but must be considered in the context of where the force is applied.

The following sections summarize experimental and computational studies of mechanophore activity as a function of: (1) force-free reactivity, (2) the geometry of attachment, and (3) the polymer backbone through which force is delivered to the mechanophore. We focus our discussion on the *gem*-dihalocyclopropanes, both because of our familiarity with this system and because quantitative data is available for all of the desired types of comparisons within this one class of mechanophores (Fig. 3), but comparative studies have been reported for other mechanophore families, and several of them are mentioned where appropriate. Regardless of the system, the results are consistent with the expectations set by eqn (2), although in some cases subtle structural effects “beyond the mechanophore” must be considered. Taken together, the molecular principles for mechanophore design are shown to be both qualitatively and quantitatively useful in a way that makes the field both attractive and accessible to mechanistic chemists.

## Effect of force-free reactivity

The change in geometry, or activation length, associated with a given reaction is a function of its mechanism. For reactions that are coupled to a force of tension through the same attachment points and that proceed *via* the same mechanism, then, the associated values of Δ*x* are expected to be similar. In such cases, those reactions that have the lower force free activation energies should require smaller forces in order to be activated on a given time scale (although this simplistic correlation has been computed to break down at very high forces for homolytic bond dissociations^31^). A homology between reaction mechanism and reaction mechanics was first noted in the scaling of the force-promoted displacement of pyridine ligands from *N*,*C*,*N*-pincer Pd(ii) complexes,^32^ but the impact of force-free activation energy on the amount of force required for activation is also evident in our chosen model system of dihalocyclopropane mechanophores.

**Fig. 4:**
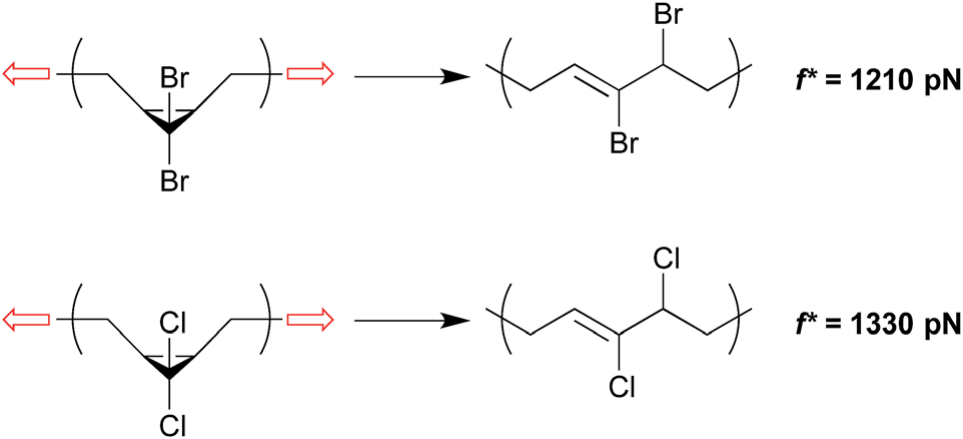
*g*DBC and *g*DCC mechanophores embedded along a poly(butadiene) backbone are activated at forces of 1210 pN and 1330 pN respectively under single molecule force spectroscopy on the time scale of ∼0.1 s.^30^ The lower force required for *g*DBC relative to *g*DCC mirrors the force-free activity.

Both *gem*-dibromocyclopropane (*g*DBC) and *gem*-dichlorocyclopropane (*g*DCC) undergo disrotatory ring opening reactions with concomitant halide migration to give the corresponding 2,3-dihaloalkene products. The mechanisms are nearly identical, but Δ*E*_act_ is ∼4.5 kcal mol^–1^ higher for *cis-g*DCC than for *cis-g*DBC.^33,34^ The forces required to achieve reaction on a given time scale should therefore be greater for *g*DCC than *g*DBC, and this is observed in single molecule force spectroscopy (SMFS) experiments.^30^ The relevant time scale for SMFS is ∼0.1 s, and the forces required to activate *g*DBC and *g*DCC mechanophores embedded along a poly(butadiene) backbone on that time scale are 1210 ± 100 pN and 1330 ± 70 pN respectively. We note that the easier activation in *g*DBC *vs. g*DCC is also observed in studies of bulk materials subjected to shear *via* extrusion^16^ and compression,^18^ although the differential activity observed might also be influenced by the differences in bulk properties of the two polymers (Fig. 4).

**Fig. 5:**
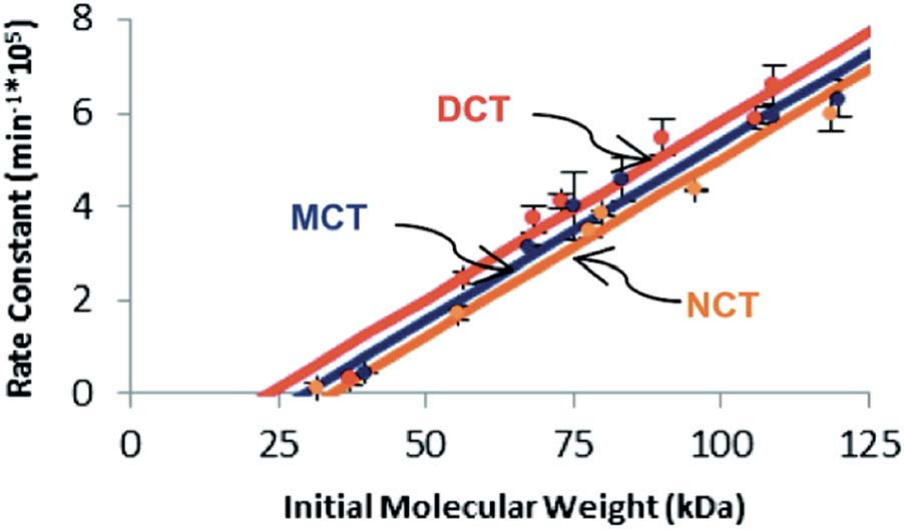
Plot of experimentally determined rate constants of polymer cleavage as a function of initial polymer molecular weight for *trans* dicyano-substituted cyclobutanes (DCT), *trans* monocyano-substituted cyclobutane (MCT), and *trans* cyclobutanes having no cyano substituents (NCT). Reprinted with permission from ref. 35. Copyright 2011 American Chemical Society.

Similar trends in reactivity have been noted in the scission of *trans*-substituted cyclobutanes *via* mechanochemically triggered retro [2 + 2] cycloadditions.^35^ When comparing the susceptibility of cyclobutanes to mechanochemical scission as a function of the number (0, 1, or 2) of cyano substituents, Kryger *et al.* found that the mechanophore requires less force for activation (as quantified by the limiting molecular weight necessary for scission to be observed on the time scale of their pulsed ultrasonication experiments) as the number of cyano groups increases, lowering the activation energy of the force-free reaction. The time scale for reaction in these experiments (∼10^–8^ s, dictated by the peak elongational strain rates) is much shorter than that in the SMFS experiments. Even at the huge forces required for reaction on this time scale, however, the trend in activity agrees with calculations of the intrinsic reactivity, in agreement with the expectations of eqn (2) (Fig. 5).

## Effect of pulling stereochemistry and regiochemistry

In addition to force-free reactivity, the activity of a mechanophore also depends heavily on the geometry changes associated with going from ground state to transition state, as captured in Δ*x*. For example, the *cis vs. trans* stereochemistry of pulling can have a significant effect on mechanical activity in electrocyclic ring openings. One effect is that the direction of pulling changes Δ*x*. A *cis* stereoisomer is initially more compact than the corresponding *trans* isomer, and so the *cis* isomer will have a greater activation length if the two proceed through a common (or structurally very similar) transition state. For example, the minimum force required at 300 K for *g*DCC activation is calculated to be 1.5 nN for the *cis* mechanophore and 2.5 nN for the *trans* mechanophore,^36^ which is in agreement with recent SMFS data.^37^ Similar trends in the reactivity of *cis*/*trans* isomers have also been noted in cyclobutane-based mechanophores.^35,38,39^

**Fig. 6:**
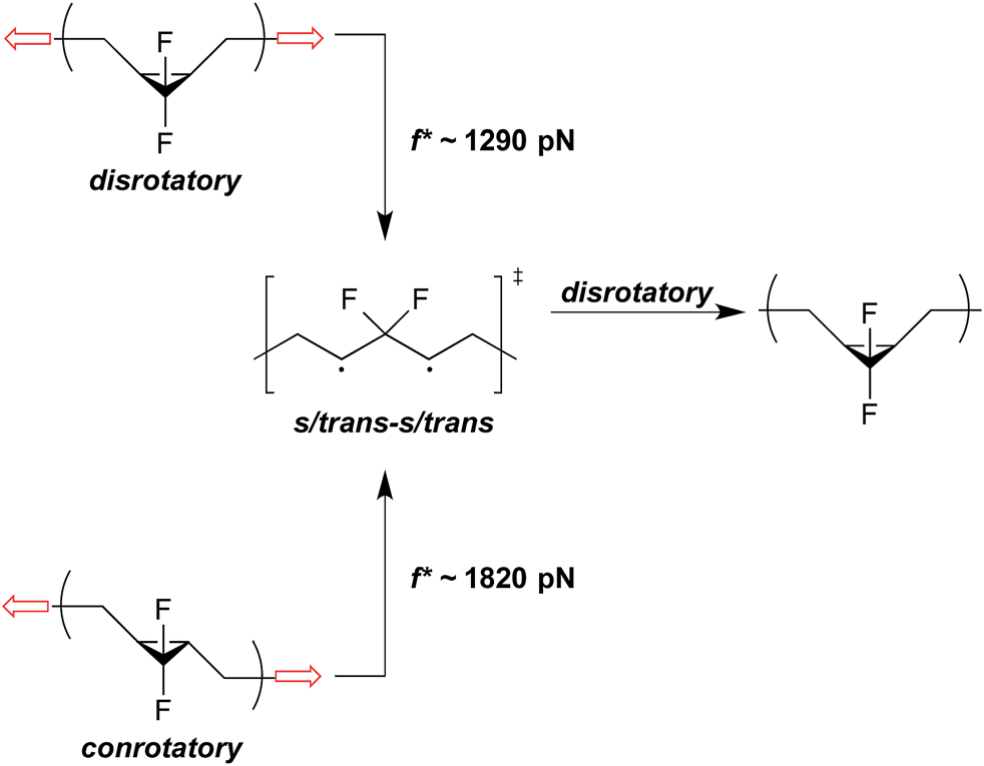
Under applied force, *cis*- and *trans-g*DFC open to the same s/*trans*-s/*trans* 1,3-diradical, which is a minimum on the force-modified potential energy surface,^22^ at f* ∼ 1290 pN and f* ∼ 1820 pN on the ∼0.1 s time scale of an SMFS experiment, respectively.^37^ When force is removed, the 1,3-diradical becomes a transition state for the disrotatory inversion path from *trans*- to *cis-g*DFC.

A second effect is that the direction of pulling can change the underlying reaction mechanism and, in doing so, have a substantial effect on the Δ*E*_act_ that must be overcome mechanically. Returning to the gDHC ring opening example, *cis* stereochemistry pulling triggers a disrotatory ring-opening that is symmetry allowed; however, *trans* stereochemistry pulling triggers a conrotatory ring opening that is symmetry forbidden.^22^ At sufficiently high forces, therefore, *trans* pulling must proceed across a higher activation barrier and do so with the lesser mechanical advantage provided by a smaller Δ*x*, relative to *cis* pulling. In the case of *gem*-difluorocyclopropanes (*g*DFCs), both the *cis*- and *trans*-stereoisomers are pulled to the same s-*trans*/s-*trans* 1,3-diradicaloid, which is a minimum on the force-modified potential energy surface (Fig. 6).^22^ SMFS reveals that this transition occurs at f* ∼ 1290 pN and f* ∼ 1820 pN for *cis-g*DFC and *trans-g*DFC respectively.^37^ Upon removal of the force, the 1,3-diradical becomes a transition state and undergoes a thermally allowed disrotatory ring closure to yield primarily the *cis* isomer, resulting in a net *trans* to *cis* mechanical isomerization.^22^ Interestingly, application of a large force of stretching results here in a polymer that actually becomes shorter, as the *cis-g*DFC has a shorter end-to-end distance than does the *trans-g*DFC.^22^

**Fig. 7:**
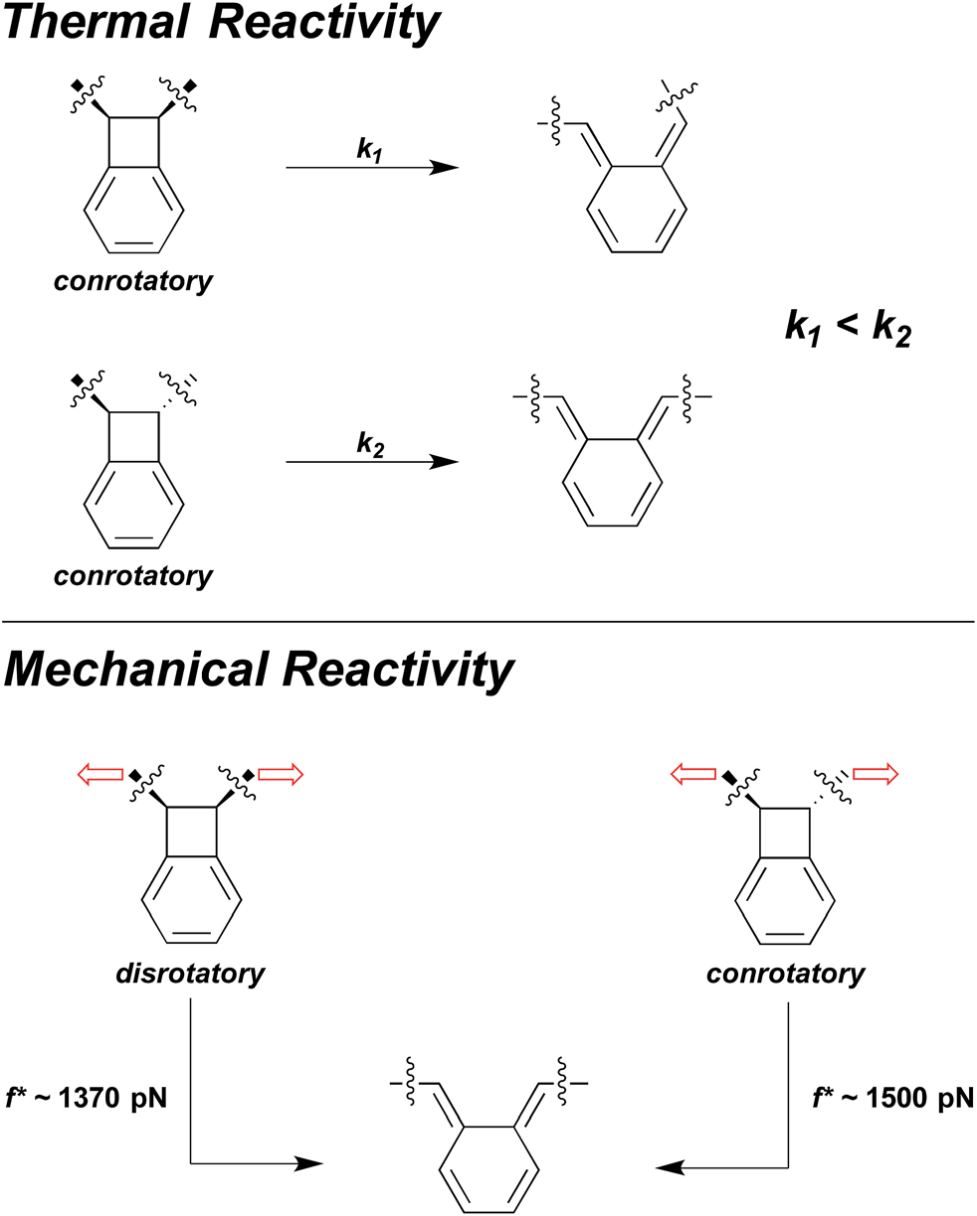
The force-free conrotatory ring opening of *trans*-BCB is much faster than the force-free conrotatory ring opening of *cis*-BCB, and yield different isomer products. But under the influence of mechanical force, the disrotatory ring-opening pathway of *cis*-BCB becomes more favourable than the conrotatory pathway^38^ and even occurs at a lower force on the ∼0.1 s time scale of SMFS experiments than the conrotatory ring-opening of *trans*-BCB.^37^

Sometimes the two effects are opposed, and at sufficiently high forces, the effect of large, coupled geometry changes will overtake the effect of lower intrinsic activation energy. For example, in benzocyclobutene (BCB) mechanophores the force-free conrotatory reaction of *trans* is much faster than the force-free conrotatory ring opening of *cis*.^38^ But, under the influence of high sonochemically generated flow forces, the *cis*-coupled isomer was found to react to a greater extent than the *trans* isomer.^38^ SMFS studies have shown that the crossover in the relative reactivity of the two isomers occurs at forces approaching 1.5 nN.^37^ These high forces do enough work on the *cis* BCB to reduce the force-coupled activation energy of the disrotatory process in the *cis* isomer to a lower value than that of the conrotatory process in the *trans* isomer, even though the former is known to be the higher energy ring-opening pathway in the absence of force (Fig. 7).^38^

In addition to stereochemical effects, regiochemical effects can also be significant. For example, a pair of computational studies by Konda *et al.* and Brantley *et al.* have suggested that the mechanical reactivity of a Diels–Alder adduct^40^ and a 1,2,3-triazole moiety^41^ can be tuned *via* strategic positioning of the attached polymer handles. In the case of the Diels–Alder adduct, pulling from the nitrogen on the maleimide and the 9-position on the anthracene result in acceleration of the cycloreversion by lowering the barrier to activation. Pulling from the nitrogen on the maleimide and the 2-position on the anthracene suppresses the cycloreversion.^40^ For the triazole moiety, Δ*x* is larger when a force is applied between the 1 and 5 positions than when the same force is applied directly between the 1 and 4 positions.^41^ Additionally, they found an increased molecular compliance along the reaction coordinate for the 1,5-disubstituted regioisomer compared to the 1,4-disubstituted regioisomer.^41^ It should be noted, however, that computational work by Smalø *et al.* suggests that, at least in the case of the 1,4-triazole moiety, the critical force required for a purely mechanical retro-[3 + 2] cycloaddition is higher than the force required to break bonds within the polymer attachments.^42^

## Mechanical coupling beyond the mechanophore

**Fig. 8:**
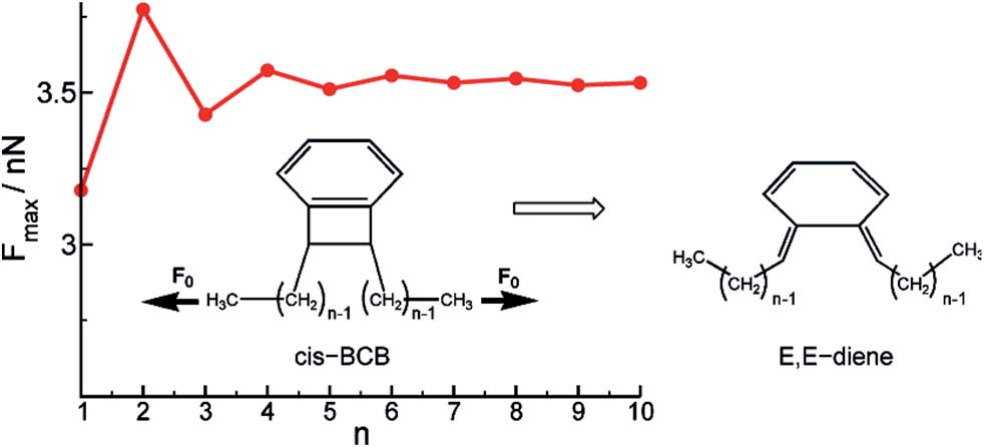
Analysis of force transduction in BCB-C_*n*_ as a function of chain length *n*. The red line shows the dependence of the breaking force *F*_max_ on the polymer length *n*. Reprinted with permission from ref. 36. Copyright 2011 American Chemical Society.

The above examples illustrate how mechanical reactivity is affected by the intrinsic activation energy, the magnitude of the applied force, and the force-coupled geometry changes associated with going from the ground state to the transition state, Δ*x*. It should be noted, however, that the definition of Δ*x* is somewhat ambiguous, in that exactly which nuclear positions determine Δ*x* is not specified. Determining where the mechanophore stops and the polymer handles begin can be difficult in many cases. For example, Ribas-Arino *et al.* determined that the rupture force (*F*_max_) of a *cis*-1,2-disubstituted benzocyclobutene (BCB) mechanophore depends on the length of polyethylene attachments (Fig. 8), and the required force does not converge until about five or six methylenes are included in the calculation.^43^ The intrinsic activation energy remains effectively constant with attachment length, and so exactly where the force is applied will have an influence on Δ*x*. This influence can be considered in the context of local structural distortions, such as the out-of-plane distortion angle *φ*(*n*),^43^ but the key point is that when force is applied through a polymeric handle, the coupling between force and reaction involves geometry changes that are felt in the polymeric handle as well, and a rigorous treatment needs to take this into account. We return to this point later in this section.

More localized structural effects of linkage on Δ*x* have been noted as well by Tian *et al.* in the force-dependent ring-opening activation free energies, Δ*G* ^‡^(*F*), of *trans*-cyclobutene derivatives with attachments consisting of a series of alkyl, ether, and ester linkages.^44^ Using density functional theory calculations, they found that Δ*G* ^‡^(*F*) is strongly affected by C5, C6 substitution (alkyl *vs.* OR *vs.* CO_2_R), but that substituents farther from the mechanophore have a much more modest effect on the force-coupled activation energy. In particular, additional force is required to produce the same barrier lowering in the diether cyclobutene series as in the dialkyl cyclobutene series. The need for this extra force was ascribed to a form of entropic elasticity needed to eliminate a subset of alkoxy conformers that are absent in the alkyl series due to destabilizing *gauche* interactions, suggesting that purely alkyl polymers are more efficient in transmitting force to the mechanophores than alkoxy substituents.^44^

**Fig. 9:**
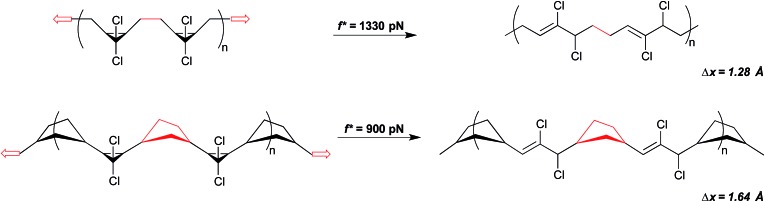
A simple change in polymer backbone from poly(butadiene) (PB) to poly(norborne) (PNB) increases Δ*x* by ∼0.3 Å in the case of *g*DCC mechanophores. The red highlighted portion of the backbone depicts how the connected atoms become misaligned with the direction of the pulling force in the case of PB, but remain aligned in the case of PNB. Note, not all *g*DCC mechanophores are adjacent as depicted.

The significance of how linkages influence mechanochemical coupling is perhaps most quantitatively demonstrated again through the *gem*-dihalocyclopropanes. Motivated by an observation that the mechanical activity of epoxide mechanophores in sonication experiments is enhanced when the epoxides are embedded along the main chain of a poly(norbornene) (PNB), as opposed to a poly(butadiene) (PB), scaffold,^45^ the backbone-related mechanical advantage was quantified in the *g*DHC polymers using SMFS.^30^ As noted above, the rate-dependent force required for the ring opening of *g*DCC and *g*DBC activation is 1210 and 1330 pN, respectively, in PB (time scale ∼ 0.1 s). But when the same mechanophores are embedded along a PNB backbone, mechanical activation occurs at 740 and 900 pN for *g*DBC and *g*DCC, respectively. For both sets of *g*DHC mechanophores, mechanical activation is observed at a lower pulling force in PNB than in PB, indicating that a change in polymer backbone can have a profound effect on mechanical reactivity. Notably, the polymer backbone effect in this system is even greater than the effect of changing the intrinsic reactivity barriers *via* the halogen (*i.e.*, chlorine to bromine). This enhanced mechanical advantage, or efficiency of mechanical force transduction through a polymer handle, is attributed to a backbone lever-arm effect (Fig. 9) that enhances the effective Δ*x*.^30^

To quantify Δ*x* for these systems, SMFS curves were fit to modified freely jointed chain models of polymer extension^27,46^ coupled to a force-accelerated transition. For both *g*DHCs, the calculated values of Δ*x* are ∼0.3 Å larger for the PNB system than the PB system, corresponding to a 10^3^-fold differential rate acceleration in the PNB polymers relative to PB at a force of 1 nN. The results are consistent with a picture in which Δ*x* is best viewed as the change in polymer contour length that accompanies the change from ground state to transition state along the reaction path of interest. Modelling the change in contour length with simple molecular mechanics force fields provides results that are quantitatively consistent with this interpretation.^30^ The origins of the lever arm effect are depicted in Fig. 9, and are relatively well communicated in a two dimensional picture of the reaction. Due to the structure of the mechanophore and the polymer, the carbon–carbon bond midway between adjacent *g*DHCs (or between a *g*DHC and an adjacent unfunctionalized PB alkene) is initially aligned almost perfectly with the vector of applied tension (the vector connecting the two ends of the polymer) along the backbone. Upon activation, however, that bond is no longer aligned with the end-to-end vector of the polymer. This bond reorientation partially offsets the lengthening expected from the local extension of the methylenes attached to the cyclopropane, and the effective Δ*x* is reduced as a result. No such effect is present in PNB, simply because of the geometry inherent in the attached cyclopentyl rings.

## Conclusions

The mechanophore concept has potential utility in stress-sensing and stress-responsive polymers, but its impact will likely depend on the ability to program mechanophores with a desired activity for a force of interest. The factors that influence reactivity are well established, and the first-order factors that influence mechanical activity (intrinsic activation energy and force-coupled geometry changes) are intuitive and accessible to synthetic chemists. This accessibility in turn lays the foundation for creativity, and the number of reported mechanophores, many with interesting and potential useful functionality, is growing at an impressive clip.^7–9,11,12,19,22,25,38,39,45,47–53^ Tuning intrinsic reactivity and the regio- and/or stereochemistry of polymeric handles has figured prominently in these advances.

Largely neglected until recently, however, is the relative importance of looking “beyond the mechanophore” in molecular design, by which we mean subsets of nuclei that are not typically considered to be directly involved in bond making/breaking. As shown in the poly(norbornenes), these so-called “lever arm effects” can have a substantial impact on activity and might ultimately be especially useful in cases where it is desirable to balance high inertness in the absence of force with good activity when force is applied. As the intrinsic reactivity and the desired force for onset of activity decrease, increasing values of Δ*x* are required, and so the ability to adjust it for a given mechanophore and reaction mechanism could be highly beneficial. That advantage is seen already in the dihalocyclopropane systems; *g*DCC embedded in PNB combines greater thermal stability and greater mechanical activity than *g*DBC embedded in PB, even though the same reaction mechanism is at play in both mechanophores. The methods by which to gauge lever arm effects in polymer mechanochemistry are also rather intuitive and easy to implement, and given their accessibility it seems likely that highly effective and reasonably general handles might be developed and applied in the near future.
